# Ischemic Colitis Secondary to Olanzapine and Clonidine Use in a Patient With a History of Laxative Abuse

**DOI:** 10.7759/cureus.36605

**Published:** 2023-03-23

**Authors:** Bosco C Theodore, Ashley Foulkrod, Priscilla Fujikawa, Kashyap Patel

**Affiliations:** 1 Psychiatry, LewisGale Medical Center, Salem, USA; 2 Internal Medicine, Edward Via College of Osteopathic Medicine (VCOM)–Virginia, Blacksburg, USA; 3 Internal Medicine, LewisGale Medical Center, Salem, USA

**Keywords:** bipolar disorder treatment, olanzapine side effects, clonidine side effects, bisacodyl, laxative abuse, ischemic colitis

## Abstract

Ischemic colitis is the most common type of intestinal ischemia and is caused by an acute arterial occlusion, thrombosis, or hypoperfusion of the mesenteric vasculature. This case centers around a 39-year-old female with a past medical history significant for a 20-year history of stimulant laxative abuse, chronic constipation, bipolar disorder, and anxiety that presented with ischemic colitis following 21 days of obstipation. At the time of presentation, the patient was taking olanzapine 15 mg daily for the treatment of bipolar disorder and clonidine 0.2 mg three times daily for anxiety. Over the course of her hospitalization, the patient was found to have a high stool burden, including calcified stool, contributing to ischemic colitis. She was successfully treated with a clonidine taper, multiple enemas, and laxatives. Pharmacological agents that induce constipation have been shown to increase the risk of colonic ischemia by increasing intraluminal pressure in the colon. Atypical antipsychotics block peripheral anticholinergic and anti-serotonergic receptors, limit gastrointestinal muscle contractions, and delay intestinal transit.

## Introduction

Intestinal ischemia results from a reduction in blood flow to a level that is inadequate for the delivery of nutrients and oxygen to the intestinal tissue. This process can be caused by an acute arterial occlusion, venous thrombosis, or hypoperfusion of the mesenteric vasculature leading to non-occlusive ischemia [1]. The incidence of intestinal ischemia is estimated to be 16 cases per 100,000 person-years [2]. Intestinal ischemia most frequently occurs in the colon and should be suspected in patients that present with crampy lower abdominal pain and hematochezia. While most cases of colonic ischemia resolve without any long-term sequelae, approximately 15% of patients will develop life-threatening consequences like necrotic bowel [2]. Here, we present a case of ischemic colitis secondary to olanzapine and clonidine use.

## Case presentation

A 39-year-old Caucasian female with a past medical history of chronic constipation, laxative abuse, bipolar disorder, anxiety, alcohol abuse, and hypothyroidism presented to the emergency room with a 21-day history of obstipation and a two-day history of constant severe sharp left lower quadrant abdominal pain, nausea, and vomiting. Before presenting to the emergency department, the patient attempted to relieve her constipation with six tablets of bisacodyl, without any relief. The patient reported a 20-year history of up to 20 bisacodyl tablets per day. Over the past few years, she reported that her laxative use had decreased to three tablets every two weeks. She usually had one bowel movement every two weeks and had noticed worsening hematochezia over the last three months, although she never sought any gastrointestinal workup. Five months prior to the presentation, she started taking clonidine 0.6 mg at bedtime to treat nighttime anxiety. The patient also endorsed taking olanzapine 15 mg daily for the treatment of bipolar disorder. It was unclear how long she had been taking the olanzapine. Olanzapine did not appear on medication reconciliations from previous hospital admissions, so it was likely a newer addition to her medication regimen.

On physical exam, she was tachycardic with a heart rate of 155 beats per minute, but vital signs were otherwise normal. Her abdomen was firm, diffusely tender, but nondistended. No guarding or rebound was noted. Laboratory values showed lactic acid (3.4 mmol/L), blood urea nitrogen (34 mg/dL), and white blood cells (15.82 x 10^3^/uL) (Table 1).

**Table 1:** Laboratory values

Lab test	Value	Reference range
White blood cells	15.82 x 10^3^/uL	4.5-10.50 x 10^3^/uL
Hemoglobin	19.1 g/dL	11.4-15.5 g/dL
Blood urea nitrogen	34 mg/dL	7-18 mg/dL
Creatinine	1.60 mg/dL	.6-1.3 mg/dL
Total bilirubin	1.8 mg/dL	.2-1.0 mg/dL
Aspartate aminotransferase	122 U/L	15-37 U/L
Alanine transaminase	185 U/L	13-61 U/L
Lactic acid	3.4 mmol/L	.4-2.0 mmol/L

**Figure 1:**
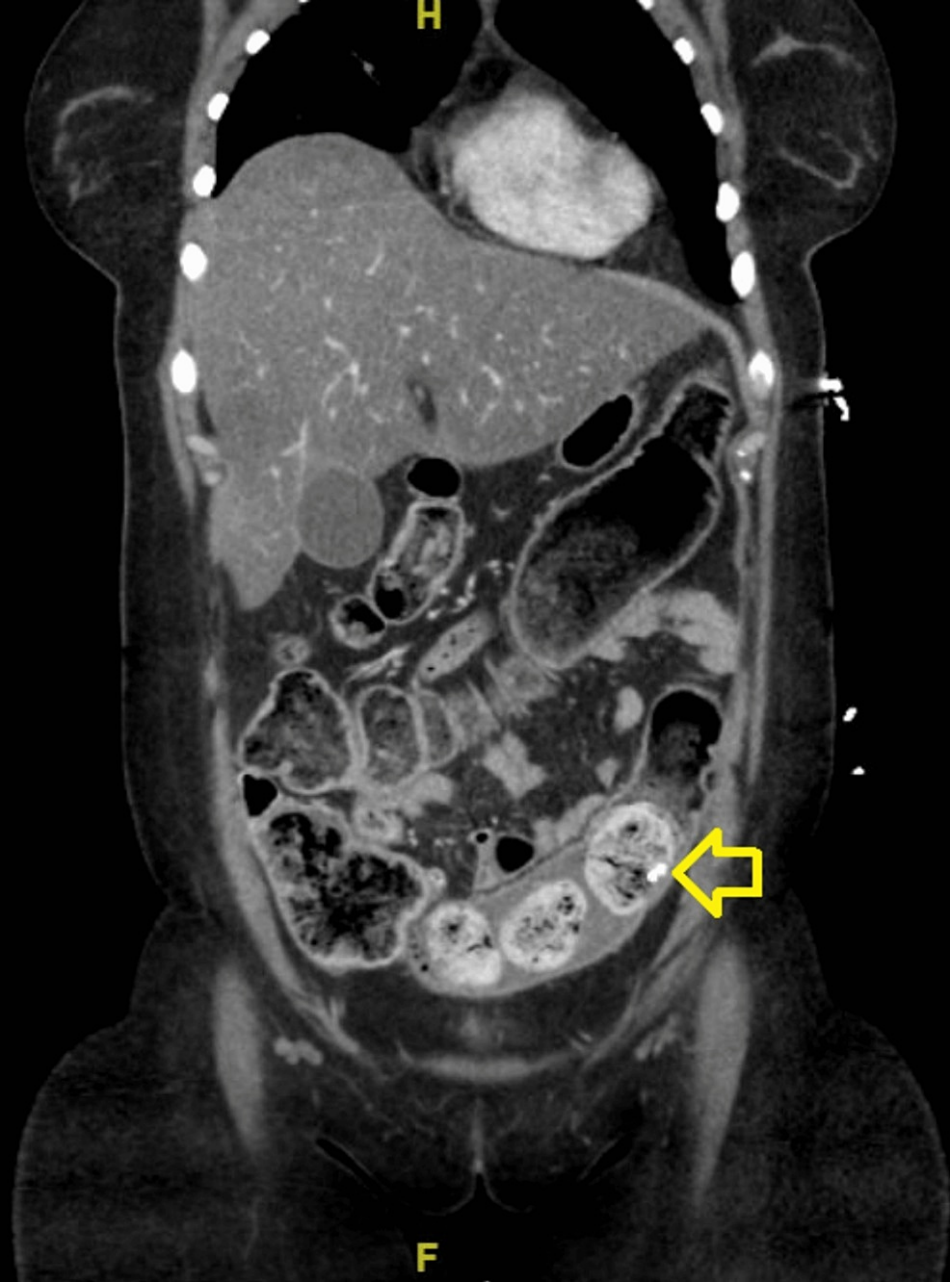
Coronal view computed tomography with intravenous contrast demonstrating a calcification in the sigmoid colon (indicated with yellow arrow)

**Figure 2:**
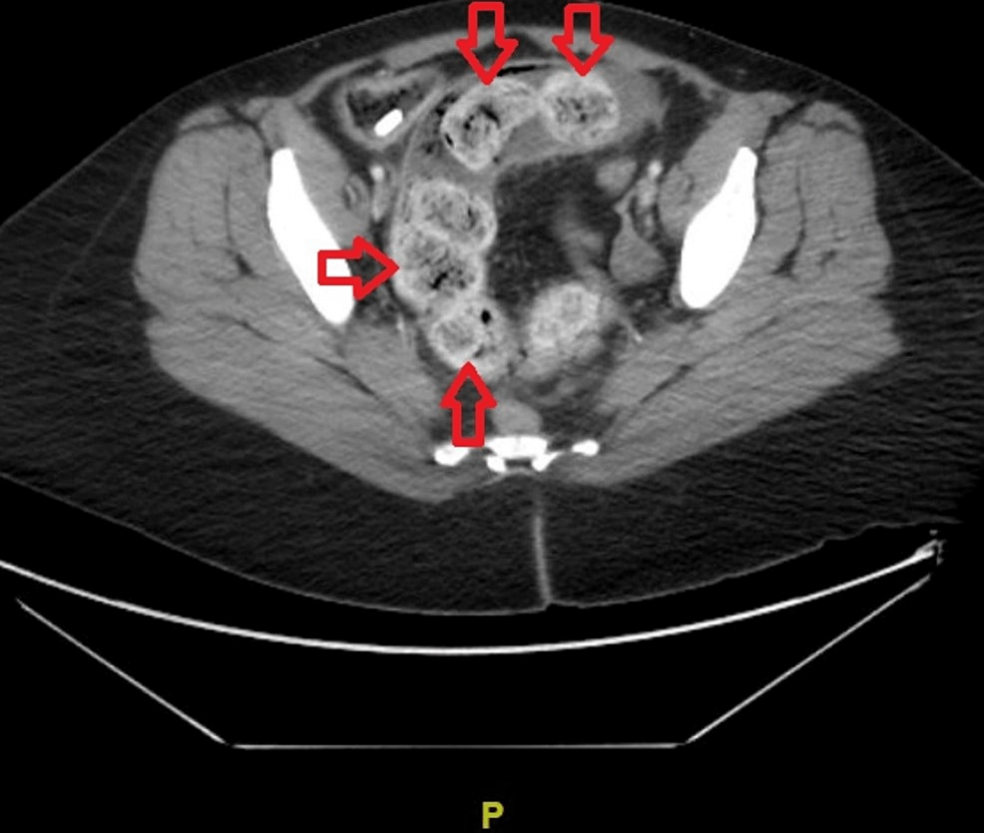
Axial view of contrast-enhanced computed tomography of the abdomen and pelvis indicating moderate to large burden of stool (Indicated by red arrows) throughout the colon

Contrast-enhanced computed tomography of the abdomen and pelvis demonstrated a calcification visualized in the sigmoid colon (Figure 1). There was a moderate to large amount of stool burden (Figure 2) throughout the colon to the rectosigmoid junction.

The gastroenterology service was consulted, and her home medications were held until she was able to tolerate oral intake. A colonoscopy on her second day of admission showed early changes consistent with ischemic colitis in the proximal sigmoid colon, involving one-quarter of its circumference. A large volume of solid stool was noted in the sigmoid colon, and the scope was no longer advanced due to the ischemic changes seen and poor bowel preparation. There was no available imaging from this procedure.

Based on the colonoscopy findings, it was concluded that her ischemic colitis was secondary to obstipation worsened by chronic laxative abuse combined with clonidine and olanzapine intake. We initiated a clonidine taper by reducing her dose by 50% every three days. Intravenous ceftriaxone and metronidazole were empirically started to protect against bacterial translocation.

Initially, a fleet enema and tap water enemas were administered, leading to three watery bowel movements. After her colonoscopy, she began to tolerate clear liquids; then, two liters of polyethylene glycol and one dose of magnesium citrate were given. She further received another two liters of polyethylene glycol, a dose of milk of magnesia, along with a gastrografin enema. Abdominal films showed a decreasing amount of stool in the colon. On multiple days of admission, the patient reported episodes of watery stool followed by increasing improvement in her abdominal pain. Her olanzapine and propranolol were held throughout her hospitalization.

The patient was discharged home with instructions to complete her clonidine taper, then advised to discontinue clonidine and propranolol. She was also instructed to consult with her psychiatrist about trialing a less constipating antipsychotic, if possible. Gastroenterology recommended the patient to continue consuming packets of polyethylene glycol orally twice daily with water for one month and then once daily for life. It was also recommended that the patient returns in six to eight weeks for a full colonoscopy; however, the patient has been lost to follow-up.

## Discussion

Ischemic colitis is the most common type of intestinal ischemia and most often affects older adults. Many classes of pharmacological agents are associated with an increased risk of colonic ischemia (Table 2) [3].

**Table 2:** Classes and examples of pharmacologic agents associated with ischemic colitis

Class	Example
Antibiotics	Amoxicillin, ampicillin, macrolides, cephalosporin, chloramphenicol, fluoroquinolones, tetracycline
Appetite suppressants	Phentermine
Chemotherapeutic agents	Vinorelbine tartrate, vinorelbine, paclitaxel, docetaxel
Constipation-inducing medications	Clozapine, voglibose
Decongestants	Pseudoephedrine
Cardiac glycosides	Digoxin, ouabain
Diuretics	Ethacrynic acid, furosemide
Ergot alkaloids	Ergotamine tartrate, methysergide
Hormonal therapies	Oral contraceptive pills
Hyperlipidemic agents	Simvastatin
Illicit drugs	Amphetamines, cocaine
Immunosuppressive agents	IL-2 agents, sodium aurothiomalate, solumedrol/azathioprine
Laxatives	Sodium polystyrene sulfonate/sorbitol, magnesium citrate/sodium phosphate, bisacodyl, glycerin enemas
Nonsteroidal anti-inflammatory agents	Meloxicam
Psychotropic medications	Amitriptyline, chlorpromazine
Serotonin agonists/antagonists	Sumatriptan, alosetron/tegaserod
Vasopressor agents	Vasopressin, glypressin

Medications that worsen constipation increase the risk of colonic ischemia by increasing the intraluminal pressure in the colon and causing blood to shunt away from mesenteric vasculature [4].

Pharmacologic risk factors associated with ischemic colitis include constipation-inducing drugs, immunomodulators, and illicit drugs. Constipating medications increase the intraluminal pressure within the colon and cause blood to be diverted away from the mesenteric vasculature, therefore increasing the risk of intestinal ischemia [1-4].

Patients that chronically use stimulant laxatives are known to have a loss of haustral folds, which suggests the presence of neuronal damage to the musculature of the colon [5]. Furthermore, colonic biopsies taken from patients with a long-term history of stimulant laxative abuse have shown that submucosal nerve fibers become damaged in a dose- and time-dependent manner. When compared to normal samples, colonic nerve endings in patients with a history of laxative abuse demonstrate a significant decrease in neurosecretory granules. The observed damages sustained to the enteric plexus likely contribute to the dysfunctional gut motility seen in patients that chronically use stimulant laxatives [6].

The development of ischemic colitis following bisacodyl administration has been reported in two patients. Both patients were young and had no past medical history but developed hematochezia and abdominal pain hours after taking bisacodyl. It was postulated that the increase in colonic motility caused a reduction in colonic mucosal perfusion [7]. Unlike the patient we presented, these patients had never taken bisacodyl or abused stimulant laxatives previously before the presentation.

There have been limited studies related to the effects of the long-term use of stimulant laxatives. It is not clear if the neural damage sustained by the colon recovers once laxatives are stopped or if their use is decreased. Additionally, there is a need for further research related to the effects of long-term purging behaviors like laxative abuse and the complex interplay with agents that limit gastric motility and induce constipation.

Olanzapine is an atypical antipsychotic with potent anticholinergic properties that have dose-dependent increases in anticholinergic activity, including constipation [8]. Constipation is a well-known side effect of olanzapine, occurring in 4%-11% of patients [9]. Numerous cases of ischemic colitis following olanzapine use have been reported [10-12].

While the exact mechanism by which antipsychotic drugs lead to ischemic colitis is not fully understood, one study reported a case of severe ischemic colitis following olanzapine 15 mg use in a 38-year-old male for five years. The authors proposed that the leading mechanism of antipsychotic-induced ischemic colitis involves the inhibition of peripheral anticholinergic and anti-serotonergic receptors, which ultimately limits gastrointestinal muscle contractions and delays intestinal transit, therefore promoting constipation. The blockage of 5-HT3 receptors reduces the gastrointestinal autonomic reflexes and sensitivity to distention, causing an increase in colonic compliance [11]. Previous studies have demonstrated that elevations in intraluminal pressure cause blood to be shunted away from the colon, therefore predisposing the colon to an ischemic event [13]. In the patient we reported in this study, her olanzapine use likely caused her to experience an increase in colonic compliance, resulting in an increase in stool burden and subsequently an elevation in intraluminal pressure. This elevation in intraluminal pressure was likely a contributing factor to the development of ischemic colitis that she sustained.

Olanzapine use has also been associated with the onset of metabolic syndromes, especially dyslipidemia and weight gain [14], which predisposes patients to the development of atherosclerotic disease. The development of atherosclerotic disease can lead to arterial thrombosis or hypoperfusion in the mesentery and eventually ischemic bowel disease [15]. This potential mechanism by which antipsychotic drugs could lead to ischemic colitis needs to be further studied.

Five months prior to her presentation to the emergency room, the patient was prescribed clonidine 0.2 mg three times daily for her anxiety. She reported that she was taking all three doses at bedtime. It was unknown if this was her prescribed dosing regimen as clonidine 0.6 mg is the upper limit of maximum daily dosing. Clonidine is an alpha2-adrenergic agonist that acts in the brainstem to activate inhibitory neurons, which results in a reduction of sympathetic outflow from the central nervous system (CNS). The most common side effects of clonidine are bradycardia and hypotension. It is also known to cause constipation in 1%-10% of patients [16]. To our knowledge, no cases of clonidine-induced ischemic colitis have been reported in the literature.

Clonidine has been shown to relax the fasting rectal and colon tone to reduce the perception of distention and pain in the colon and rectum in a dose-dependent manner. This alteration of tone and compliance of the colon has been shown to reduce the sensitivity to intraluminal mechanical stimuli [17]. The clonidine likely caused colonic relaxation and therefore an increase in stool burden. The clonidine also likely caused a reduction in her perception of distention and pain in the colon, which diminished her ability to feel a strong urge to defecate.

## Conclusions

In conclusion, clinicians should be aware of the medications that may predispose patients to ischemic colitis and should also consider the cumulative burden of constipating medications as their effects appear to be additive, especially in this patient with a long-term history of stimulant laxative abuse. Previous case reports have shown an association between the use of olanzapine and the development of ischemic colitis. Clonidine and chronic laxative abuse have not been reported as direct causes of ischemic colitis in the literature, although these factors likely contributed to the development of the patient’s severe obstipation and eventual ischemic colitis.
